# Tracking progress via clinical feedback systems in treatment of substance use disorders: a qualitative study exploring patients’ response processes

**DOI:** 10.1186/s40359-025-03377-6

**Published:** 2025-09-29

**Authors:** Øyvind Grindheim, Heidi Brattland, Christian Moltu, Andrew McAleavey, Kristin Tømmervik, Hege Govasmark, Valentina Iversen

**Affiliations:** 1 Department of Mental Health, Norwegian University of Science and Technology, Trondheim, Norway; 2 Nidelv Mental Health Center, St. Olavs University Hospital, Trondheim, Norway; 3 Department of Psychology, Norwegian University of Science and Technology, Trondheim, Norway; 4 District General Hospital of Førde, Førde, Norway; 5 Department of Health and Caring Sciences, Western Norway University of Applied Science, Førde, Norway; 6 Weill Cornell Medical College, New York, NY USA; 7 Clinic of Substance Use and Addiction Medicine, St. Olavs University Hospital, Trondheim, Norway; 8 NTNU Department of Mental Health, Trondheim, NO-7491 Norway

**Keywords:** Routine outcome monitoring, Feedback system, Substance use disorders, Response processes, Cognitive interviewing, Qualitative research

## Abstract

**Background:**

In recent decades, there has been a shift in many substance use disorder (SUD) treatment settings toward routine outcome monitoring via clinical feedback systems (ROM/CFS). This move toward frequent measurement throughout the treatment course raises new questions about which variables should be measured in the evolving treatment process. In particular, there seems to be a lack of knowledge concerning self-report measures’ ability to capture patients’ subjective experiences relevant for treatment outcome and process. This qualitative study explored patients’ perspectives and reasoning processes to investigate how feedback systems can be adapted to track progress in the treatment of SUDs.

**Methods:**

Patients (*N* = 13) in specialized SUD treatment were interviewed while they responded to items from a feedback system used for routine outcome monitoring in that treatment setting. The approach to data collection was inspired by cognitive interviewing and the analysis was based on a descriptive and interpretative approach.

**Results:**

The findings provide support for monitoring of several outcome variables, such as behavioral and experiential symptoms, as well as variables relevant to everyday functioning. However, we also found that several aspects related to the subjective experience of being in an ongoing process of change provided important context for the various outcome variables during treatment. The analysis resulted in three themes reflecting this duality and issues that appeared important to consider together when adapting clinical feedback systems to longitudinal monitoring in SUD treatment: 1) The necessity of capturing both preliminary outcomes and the attitude toward the ongoing process of change; 2) The necessity of capturing both the passive and the active role of the patient; and 3) The necessity of capturing both objective and subjective change constructs. Related to each theme we describe the participants’ different interpretations of specific items and how different item characteristics facilitated meaningful measurement or not.

**Conclusions:**

The findings expand the knowledge base and strengthen the qualitative foundation for longitudinal monitoring of SUDs and have implications for how feedback measures should be designed. The study also provides knowledge about the process of change and highlights patient experiences that are worth paying attention to when tracking progress in treatment of SUDs.

## Introduction

In recent decades there has been a shift in many substance use disorder (SUD) treatment settings toward routine outcome monitoring via clinical feedback systems (ROM/CFS) [1–4]. ROM/CFS involves the routine collection of treatment-relevant information via patient self-reports. This information is fed back to therapists so that treatment can be adapted according to the individual patient’s needs [5]. Preliminary findings indicate that the use of feedback systems can improve outcomes in SUD treatment, especially for patients who do not progress as expected [4, 6]. However, the shift toward more frequent measurements during treatment also raises new questions regarding which variables to measure in the evolving treatment process.

Questions about what to measure, when, and how may be particularly important to address for feedback systems used in the treatment of SUDs, because ROM/CFS has primarily been developed and research on within mental health treatment settings [2]. SUDs tend to be long-term conditions associated with severe functional impairment and with treatment trajectories often characterized by dropout and relapse [7–9]. The unpredictable and fluctuating course of SUDs makes progress monitoring a complex task in many cases and underscores the importance of having a system that can support patients and clinicians in tracking progress and tailoring interventions during treatment.

Traditionally, progress in SUD treatment has been evaluated based on treatment attendance, urine drug screens and breathalyzers [2]. Over time, a number of self-report measures have also been developed that target patient experiences of SUD specific symptoms, such as the Alcohol Use Disorders Identification Test (AUDIT), the Drug Use Disorder Identification Test (DUDIT), and the Addiction Severity Index (ASI) [10–12]. Additionally, in more recent years, measures focused on long-term recovery from SUD have been developed focusing more on the long-term outcomes and the recovery process [13–15].

Despite these developments, certain issues central for longitudinal monitoring of SUDs still appear too little investigated. While there appears to be broad consensus that assessment and monitoring of SUDs should move beyond SUD-specific symptoms and include items relevant for personal and social recovery [14, 16, 17], it is less clear how progress can be monitored during the course of treatment. In particular, there seems to be a lack of knowledge concerning self-report measures’ ability to capture patients’ subjective experiences relevant for treatment outcome and process. In keeping with this, service users report that the self-report measures used to track progress in SUD-treatment often fall short in following their subjective experiences and progress [18–20].

To address the abovementioned issues, we argue that increasing our understanding of patients’ response processes when responding to feedback measures in specific treatment contexts is necessary [21]. Response processes can be defined as *the mechanisms that underlie what people do*,* think*,* or feel when interacting with*,* and responding to the item or task*,* and are responsible for generating observed test score variation* [22]. The study of response processes has largely been overlooked in previous research [23] but may be key to understanding what is needed to track individual trajectories in SUD treatment.

Cognitive interviewing [24] is an approach that has been proven valuable to improve validity of self-report measures in SUD treatment [25]. The approach involves interviewing participants about their response processes while they are responding to items from a given self-report measure, either by encouraging the participants to vocalize their thought process or asking more specific probing questions. Sometimes also called cognitive testing, cognitive interviewing is typically used for pretesting of items when developing new self-report measures. The testing will then focus on how well the items capture some predefined constructs and focus on identifying and reducing measurement errors arising from participants misinterpretations of the items’ intended meaning.

However, cognitive interviewing may also be used to build knowledge bottom-up and to increase understanding about the responders’ perspective and their subjective reality and thereby increase our understanding of constructs and phenomena relevant for longitudinal monitoring. A closer elaboration of patients’ response processes on SUD-specific items, may increase the understanding of what factors are relevant to monitor in SUD treatment seen from a patient’s point of view, and how these factors can be addressed and captured via patients’ self-report, thus, contributing to an improved qualitative foundation for ROM/CFS in treatment of SUDs.

On the basis of the exploration of patients’ response processes, this study aimed to identify factors that are important for the longitudinal monitoring in the treatment SUDs and investigate how items on self-report measures can be adapted to capture treatment-relevant factors effectively via feedback measures in routine outcome monitoring.

## Methods

### Design

In this qualitative study, we interviewed participants who had current or recent experience with receiving specialized SUD treatment. The participants were interviewed while they responded to items from a clinical feedback system used for routine outcome monitoring in that treatment setting. The data collection was inspired by cognitive interviewing, including both the think aloud technique and probing questions [24]. The interviews have previously been used in a study investigating response processes for patients providing quantitative self-report [21]. In comparison, this study focused on the specific content of the patients’ reasoning process when responding to SUD-related items. In contrast to many other studies that use cognitive interviewing to compare the responder’s interpretations to a “correct” understanding of the items, we were primarily interested in describing the participants’ idiographic interpretations, thought processes, and associations triggered by the different items in the feedback system targeting SUD-specific issues. Furthermore, following a hermeneutical approach [26], we were interested in how the participants interacted with the items to make sense of them, how the individual participants’ treatment contexts influenced their responses, and how item characteristics influenced the participants’ ability to convey meaningful information.

### Materials

NORSE feedback (NF) is a feedback system used for routine outcome monitoring that was developed through a continues quality improvement project [27]. NF 2, used in this study, contains a pool of more than 100 clinical items. The items are self-evaluative statements on which respondents rate themselves on a seven-point Likert scale. Scores are provided for 19 clinical and resource dimensions [28]. When used clinically, most of the items are administered before the first session, but for subsequent sessions, only trigger items are administered at each assessment occasion, and the full scales are reopened only if scores fall above the cutoff. The NF was developed primarily for mental health specialist services, but it also includes items targeting SUD symptoms and recovery.

For the interviews, we selected items from the NF system to cover several areas to obtain a general impression of the participants’ response processes but prioritized for the purpose of this study items related to SUDs and recovery. The SUD-related NF items that were included in the interviews are presented in Table 1.

**Table 1:** Norse feedback items related to SUD symptoms and recovery

*I think that I need to cut down on my drinking/drug use*	
*I am concerned that I am dependent on drinking/drugs*	
*Other people are worried about my use of alcohol/use of drugs*	
*My use of alcohol or drugs interferes with my ability to function (in my job*,* as a student*,* as a parent*,* etc.)*	
*I believe that I can handle events in my life without drugs/alcohol*	
*I can manage a day without alcohol/drugs*	
*I am on the right path for not having drug-related problems*	

### Recruitment

Participants were recruited from March to December 2023. We recruited adult participants who were currently or had recently received specialized SUD treatment. There were no exclusion criteria. Participants were recruited from units that had implemented the NF system. Because we wanted a varied sample, we recruited from both outpatient and inpatient settings. We also wanted participants who were in different phases of treatment and had different levels of experience with using feedback measures (including the NF system) in treatment. To evaluate the sample size needed, we compared new incoming data to the existing material from the previous interviews and stopped recruiting when the last three interviews did not add significantly new information compared to the existing data material.

### Participants

A total of 13 participants were recruited, ten from outpatient and three from inpatient treatment settings. The participants’ age ranged from 22 to 72 years (*M = 48.2*,* SD = 14.8*). Five participants identified as male and eight identified as female. Among the 13 participants, six were currently employed, five received disability benefits, and two were retired. The types of substance abuse included alcohol overall/only (*n* = 8/6), cocaine (*n* = 2), amphetamine, opioids, sedatives, cannabis, and unspecified (all *n* = 1). The duration of substance use disorders ranged from 0 to 45 years (*M = 12.15*,* SD = 15.01*). In total, eight participants reported comorbid mental health disorders, including mood disorders (*n* = 4), anxiety (*n* = 2), posttraumatic stress disorder (*n* = 2), and eating disorders (*n* = 1). The duration of experience with NF ranged from 0 to 18 months (*M = 6.15*,* SD = 5.32*).

### Data collection

The participants were interviewed individually while they responded to the selected items from the NF feedback system. They were instructed to imagine that they were responding to these items as part of their treatment. The participants were then presented with the items one by one and encouraged to describe their thought processes. Additionally, they were asked probing questions to gain deeper insights into their responses and to obtain an impression of their experiences with responding to the specific items over time (*see interview guide*). The interviews focused on each patient’s response process related to the different items and especially how they made sense of and reasoned about issues related to their SUDs. The interviews were audio recorded and transcribed verbatim.

### Analysis

In the analysis, we followed principles for a descriptive interpretative approach as outlined by Elliot and Timulak [29]. This is a generic approach, that is well suited to incorporate interpretative elements in the analysis while keeping it grounded in descriptive data. The analysis included both coding and categorization of the transcribed interviews. The first coding process focused on identifying different meaning units in the patient’s response processes, typically reflecting the participants’ different thoughts and reactions when they encountered the different items. In the next step, we compared the codes to identify broader patterns of meaning or categories, for example, problems that several participants reported, or item characteristics that were helped or hindered the response process. To summarize the categories, we developed themes to describe central meaning patterns that were relevant for the research question. At this point, we also assessed the meaning patterns against the information about where each participant was in their treatment process to explore how this might have influenced their reasoning.

Because the items targeted SUD-related problems and recovery, we expected the results to concern SUD-specific symptoms and recovery. However, the analysis aimed, through the exploration of the participants’ response processes, to uncover factors and issues within and across these domains that are central for longitudinal monitoring of SUDs, and to investigate how well these factors were captured by the different items (see Table 2 in the result section for an overview of the different levels in the analytic process, including examples of meaning units and categories).

### Reflexivity

All the authors embarked on this project with a generally positive attitude toward the potential of self-report measures to monitor SUD treatment processes. However, all authors are also part of a project researching challenges, pitfalls, and necessary developments for such systems. To counter biases in implementing this qualitative study, a particular focus was placed on researcher reflexivity [30]. At the end of each interview, the interviewer summarized the main content of the interview together with the participant to check shared understandings and clarify ambiguities. Transcription, coding for deconstruction, and writing memos were also steps to promote reflexivity. Moreover, the codes, categories and themes were discussed in analytic meetings with ØG, HB, CM, AM and VI, and all coauthors read the draft manuscripts and commented on its clarity before final writing up.

### Ethical approval

The study was approved by the Regional Ethical Committee for Medical Research in Norway (application #2022/249409). All participation was based on fully informed written consent, which was signed prior to participation.

## Results

The data material reflected how the participants made sense of the different specific items. However, the interviews also provided information about the participants’ thoughts, associations and reasoning beyond what the specific items targeted and identified dilemmas that the participants encountered in their response processes. A central pattern that emerged during the analysis was a duality in the participants’ reasoning between reporting outcomes and being in an ongoing forward-looking process that was still open and undetermined.

**Fig. 1:**
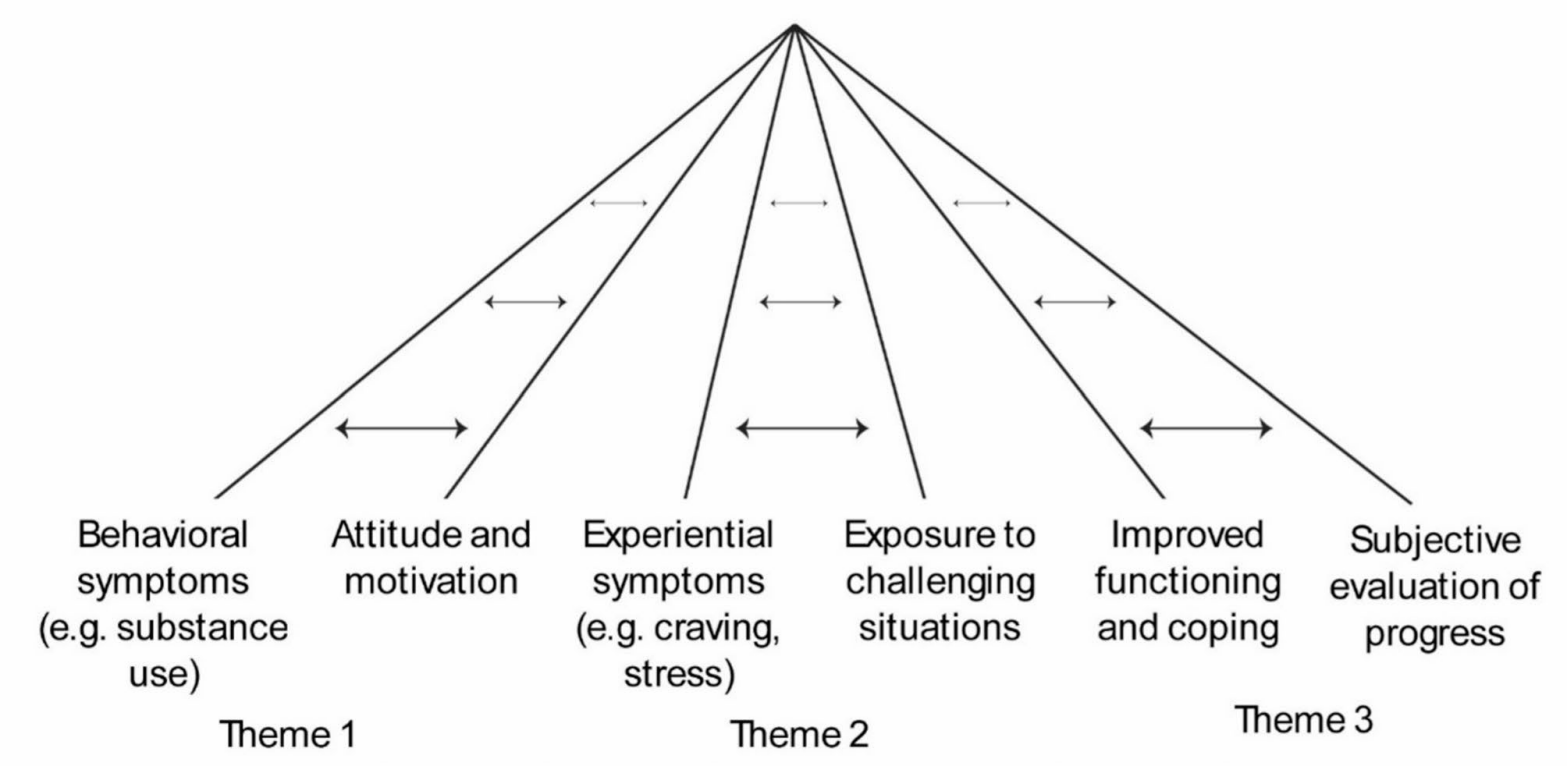
Factors that should be monitored and evaluated together when tracking progress

Our analyses resulted in three themes that reflect this duality and summarize the meaning patterns that we found particularly central for longitudinal monitoring of SUDs. For each theme, we describe commonalities and differences in the participants’ interpretations related to specific NF items. To highlight possible developments in the response process across time with repeated measurements we found it useful to distinguish between patients in an early treatment phase (approximately the first two months) and those in later phases of treatment. In addition, because the data material reflected the participants’ reasoning beyond the specific items it also revealed issues that the items were not able to capture. When relevant, we describe how various items characteristics facilitate meaningful measurement. Figure 1 provides a visual overview of the factors that were identified as important to monitor and consider related to substance use disorders. The arrows between the lines indicate outcome and processes variables that should be monitored and evaluated together.

### Theme 1: the necessity of capturing both preliminary outcomes and the attitude toward the ongoing process of change

In response to the item ‘I think that I need to cut down on my drinking/drug use’, participants in the early phase of treatment evaluated their current or recent substance use. Since most of the participants at this point had started to realize that their substance use was problematic and something they needed to change, this harmonized well with the statement in the early phase of treatment. “*I know that I need to cut down”* (P12).

Later in treatment, however, many participants reported dilemmas about how to rate the same item. Since most participants by then had managed to stay abstinent for some time, it was important for them to distance themselves from past use. On the other hand, giving a low rating on the item “I think that I need to cut down on my drinking/drug use” would give a wrong impression that the issue was no longer a problem. *“It is not something you can just leave behind and then it’s gone. It will always be there”* (P4).

Consequently, participants later in treatment ended up with different ways of interpreting and answering the item concerning their need to reduce their substance use. Most participants who managed to stay abstinent gradually found it natural to focus only on substance use during the previous week. Others would, however, continue to include past use when responding. *“I have ended up answering seven anyway*,* because I have assumed that it is kind of the situation in total considering my substance use before I was admitted”* (P11). However, another group tried to encompass both the preliminary outcome and their attitude toward the problem. “*On this I would respond with a five… almost six immediately. Because it has now been several weeks since I have tasted alcohol. Therefore*,* I kind of answer from different points of view”* (P3).

These dilemmas and different interpretations notwithstanding, several of the participants in later phases of treatment said that routinely reporting on substance use had an important function because it maintained focus and awareness and signified that they stuck to their goal of staying abstinent. At the same time, they needed to convey that they still had a vulnerability and a problem they needed to continue to work on. The item “I am concerned that I am dependent on drinking and drugs” was close to having such a complementary function, as it allowed for the participants to express concerns about their own vulnerability. However, this item was also limited by the coupling of the concern to being dependent, as this for most referred to a condition that they already knew they had, rather than a concern for their ongoing change process. *“It is not something I am worried about; I know that I am [dependent]*” (P4). Accordingly, some of the participants commented that items more directly targeting their motivation to continue working on the problem and their concern about relapse would have been preferable.

### Theme 2: the necessity of capturing both the passive and the active role of the patient

When discussing their experiences with responding to ROM/CFS items, important insights started to emerge for the participants into how symptoms, needs, and their way of coping had changed throughout the treatment process. In the early phases of treatment, some of the participants described how thoughts about substance use tended to take up much of their time. “*It [thinking about substance use] used to occupy much space*,* then it took up less space as time went by”* (P4). Substance-related cravings were also particularly prominent and mentioned by all participants in some way or another. “*I have a lot of psychological abstinence*,* and in a way that becomes psychological restlessness and discomfort”* (P11). In addition, they described states and situations that triggered and exaggerated substance-related thoughts and feelings. “*Then there are those triggers that kick in”* (P13). Typical examples of triggers could be stress, thought pressure, feeling bored, being alone, and going to places associated with drug use.

Throughout the course of treatment such passively experienced symptoms continued to be relevant to monitor. However, what symptoms the participants experienced, and the strength of those symptoms were also highly influenced by how much the participants exposed themselves to challenging situations. In particular for participants who were in later phases of treatment, taking initiative and actively seeking out new situations was an unavoidable part of the change process. Many participants described how they had become increasingly aware that they had to actively seek out situations that were challenging, yet necessary to live the life they wanted. “*When you quit using drugs*,* you also cut out a large part of your life. In addition*,* it takes a long time before you truly understand what that means or what you are exposing yourself to […] and everything you should not expose yourself for”* (P2). This increasingly future- and goal-oriented state of mind also implied a qualitative shift in the participants’ perspectives, in which they became more aware of how they had to balance the exposure to new situations so as not to be overwhelmed and how they needed to take precautions. *“I must be careful*,* especially when we are going out and such. I have things I need to watch out for… related to living an active life again”* (P9).

Few of the selected NF items targeted this active role of the patient directly. Instead, the items tended to focus on passive experiences. In consequence, several of the participants reported thoughts about how context and their own actions had influenced their symptoms during the previous week without them being able to communicate this through the feedback measure.

### Theme 3: the necessity of capturing both objective and subjective change constructs

In encountering items targeting progress, several participants described having felt ambivalent about change early in treatment. *To begin with*,* it was like why on earth should I quit?* (P9). P12 described how to begin with the only indicator of improvement was the still short-lived abstinence. “*It has improved in the sense that I have not used drugs”* (P12). Abstinence did not, however, guarantee long-term improvement, and the participants reported that it had been challenging to remain hopeful and believe that things would improve. *I don’t know if I will improve by becoming abstinent when I struggle with so many other things* (P12). Consequently, subjective change constructs, such as motivation, hope, and belief in improvement, were described as relevant indicators of change in this early phase.

Later in the treatment process, the participants tended to be more concerned with seeing actual changes in how they functioned in everyday life. In particular, reflecting on how they have coped with certain situations in the recent past appeared to be important for their self-evaluations and for addressing the root cause of their SUD. “*I started using substances because I couldn’t cope with my life*” (P3). Consequently, items that captured specific coping behaviors and exemplified improved functioning in everyday life became more relevant. *I know how it would have been if I had been drinking […] However*,* I have no financial problems*,* I’m not sick*,* my apartment looks alright*,* and neighbors greet me and all of that”* (P9).

In contrast to the responses referring to actual changes in everyday life, the participants’ responses to the item ‘I am on the right path for not having drug-related problems’ tended to reflect a greater overall experience of improvement. Compared with the items targeting more specific outcomes, this item appeared to allow the participants to take one step back and consider change across time, and their reasoning would typically include two components: one emphasizing improvement and the other the remaining needs. “*I’ve come a long way but still have some distance left”* (P1).

While the NF item ‘I can manage a day without drugs/alcohol’ was found by some participants to be too broad and hypothetical, the item ‘I believe that I can handle events in my life without using drugs/alcohol’ was more likely to trigger thoughts about specific events and past coping behavior. “*The wording ‘events in my life’ makes it easier to reason around”* (P1). In comparison, the item asking if they felt they were on the right path typically required less detailed reasoning. However, many participants appreciated this item because it was an opportunity to report a more general feeling of progress, and as one participant remarked, lower ratings on this item would be an effective way to signal to the therapist if something needed to be discussed.

Table 2 provides an overview of the different levels in the analytic process, including examples of meaning units and broader patterns of meaning associated with the items and the resulting themes.

**Table 2:** The different levels in the analytic process

Items	Examples of codes/ meaning units	Examples of categories/ patterns for meaning	Themes
*I think that I need to cut down on my drinking/drug use * *I am concerned that I am dependent on drinking/drugs * *Other people are worried about my use of alcohol/use of drugs * *My use of alcohol or drugs interferes with my ability to function (in my job*,* as a student*,* as a parent*,* etc.) * *I believe that I can handle events in my life without drugs/alcohol * *I can manage a day without alcohol/drugs * *I am on the right path for not having drug-related problems*	Reporting current substance use How problematic is it? Important reminder It will always be there No longer worried, because I have things that I am working on Holding back because I am still uncertain about the future Still a vulnerability even though I have been abstinent Worry about relapse ------------------------------ Think about it almost all the time” Takes up less time now Discomfort, restlessness, stress, craving Feels dishonest to say that I can cope with events in my life when it still affects my life so much Things to look out for (triggers, avoidance and self-protection) ------------------------------ Evaluating own progress It’s a question that makes me stop to evaluate how I am doing Abstinence may be the only indicator of change for those early in treatment Think about the things I have accomplished The formulations events in life makes it easier to resonate about specific incidences	Quantification of behavioral symptoms (substance use) Dilemma between emphasizing current use or past use Relevant issues depend on where the patient is in the process of change The attitude towards the problem and the ongoing process of change continues to be relevant (insight, motivation) Quantification of experiential symptoms (e.g. craving, stress) Exposure to difficult situations, coping, and pursuing life goal becomes more important The reasoning involves several considerations that influence symptom severity Indicators of progress changes through the treatment course May think about specific events, examples of failure or improved functioning Subjective evaluation of own change process (hopes, beliefs, goal achievements)	The necessity of capturing both preliminary outcomes and the attitude towards the ongoing process of change The necessity of capturing both the passive and the active role of the patient The necessity of capturing both objective and subjective change constructs

## Discussion

The value of feedback systems depends on their ability to capture treatment-relevant variables throughout the treatment course, but it is uncertain how well existing measurement systems are able to do so. In this study, we therefore explored patients’ interpretations and response processes when they responded to items from a clinical feedback system used for routine outcome monitoring to identify issues that are important to consider when tracking progress via clinical feedback systems in specialized treatment of SUDs.

In the analysis, we identified several factors related to behavioral, experiential and functional outcomes that continued to be relevant throughout the treatment course. However, central in our findings was a duality indicating that certain process variables, such as participants’ current attitudes, actions and subjective change constructs, provided important context for the outcome variables. The analysis thus resulted in three themes reflecting outcome and process variables that should be monitored and evaluated together: (1) The necessity of capturing both preliminary outcomes and the attitude towards the ongoing change process; (2) The necessity of capturing both the passive and the active role of the patient; and (3) The necessity of capturing both objective and subjective change constructs.

The findings highlight that each patient is in their own individual process and use their context and what they want to achieve when making sense of individual items. Their meaning-making process related to specific items may therefore depend on where they are in their treatment process. Moreover, the findings suggest that people responding to feedback measures try to convey a coherent representation of themselves and that they need different aspects to be reflected. For example, maintaining motivation to continue working on the problem was important for how the current behavioral outcome should be understood. Similarly, the participants’ willingness to expose themselves to challenging situations provided important context for their symptom experience. Finally, the participants’ subjective feelings of change and belief in treatment supplemented items targeting external indicators of change.

As expected, many of the factors identified in this study correspond with factors described in earlier research. For instance, core symptoms such as substance use and craving have long been recognized as central variables to capture in SUD assessment [31, 32]. The importance of patients attitudes toward the problem emphasized in the first theme is similar to the concept of decisional balance in the Transtheoretical Model of Change [33]. Furthermore, the issues related to progress resemble some of the themes of Neale et al. [18] who described service users’ view of measuring addiction recovery, such as that goal-oriented behaviors can create new pressures and the importance of coping rather than cure.

The more specific contribution of this study relates to the exploration of patients’ response processes and the implications for how items can be adapted to capture central issues effectively and meaningfully throughout treatment. While traditional patient-reported measures tend to be problem-oriented and emphasize symptom reduction, patient’s subjective experience tends to be more process- and goal-oriented [34]. From this perspective, being in treatment will be more like an ongoing adaptive process, continuously influenced by the person’s choices of action and context, as well as self-evaluative dimensions, such as acceptance and uncertainty. The exploration of the response processes in the present study highlights how these different aspects need to be measured and considered together in order to understand the dynamics in patients’ process of change.

The exploration of the response processes further indicated that patients may have different concerns in different stages of the treatment process. These variations may have implications for longitudinal monitoring, particularly in terms of tailoring assessment and interventions to align with patients’ evolving needs over time. Theoretical models like the Stages of Change in the Transtheoretical Model [33] and the Phase Model of Psychotherapy Outcome [35], may offer valuable frameworks for understanding what should be measured and when. Drawing on these models have the advantages that they indicate a logical sequence of change, identify necessary preconditions for progress from one stage to the next, and inform the selection of appropriate intervention strategies. However, limitations with theoretical change models relate to their ability to represent individual variations and fluctuations in patients’ change processes. Moreover, the primary focus on change, may underrepresent what is arguably a main challenge for many patients in SUD treatment – the ability to sustain positive changes over time. These specific issues may also reflect limitations in current feedback systems employed for longitudinal monitoring in SUD treatment.

Although many patients achieve abstinence and reductions in psychological distress during the initial phase of treatment, the majority of patients in SUD treatment will typically experience relapses and fluctuating symptom trajectories over extended periods before managing to stabilize positive changes [9, 36, 37]. Thus, after an initial phase, often marked by remoralization and symptom improvement, patients in later stages of treatment may find themselves in prolonged situations in which signs of progress become less evident. In this absence of clear progress, it may become increasingly important for feedback system used for longitudinal monitoring to expand focus to also encompass factors related to the patients’ ongoing efforts to stabilize changes. For example, maintaining awareness of the problems that still need to be worked on, adjusting activity levels based on current symptom intensity, and focusing on subjective experienced change constructs can be crucial when other clear indicators of progress are limited.

Incorporating items targeting process variables such as the patient’s current attitudes towards the problem, the active role of the patients, and subjective change constructs, as suggested in this study, may improve feedback systems ability to track individual progress. However, it remains uncertain to what degree self-report measures can reliably capture subjective experiences and track individual progress. Despite the availability of many different self-report measures, few have been specifically developed and validated for longitudinal monitoring [4, 32]. Given the various and context-dependent nature of change for patients in SUD treatment, self-report alone is likely insufficient to capture nuanced and in-depth qualitative information about the subjective experiences that underlie and motivate patients’ responses and progress. A key strength of ROM/CFS thus lies in its feedback component, facilitating an informed dialogue between patient and therapist about how reported experiences unfold in daily life.

The findings in this study may improve feedback systems’ ability to capture patients’ subjective experiences and individual change processes. However, the study focused on a small number of SUD-specific items. Hence, the findings should be considered together with research focusing on a broader scope of outcomes, in particular research focusing on SUD assessment [38] and recovery [17, 18, 39]. Moreover, the findings should be seen together with research focused on ideographic ROM/CFS and goal-based outcomes, which also emphasize service users’ perspective and subjective change constructs [40, 41].

### Implications

The duality between outcome and process identified in this study suggest that feedback measures used in psychologically based SUD treatment should include both items targeting outcomes and items targeting thoughts, feelings and actions related the ongoing process of change. The first and second theme highlight that assessment of symptoms may advantageously be accompanied by questions focusing on the person’s attitude, engagement, or motivation to continue to work on the problem, as well as how much they have challenged themselves. The third theme suggest that it is relevant for feedback systems to include items targeting both external indicators of change, as well as items targeting more subjective chance constructs such as hope of improvement or feelings of being on the right track.

The findings highlight that when tracking progress via feedback system it is important to provide patients with the opportunity to express both progress and concerns. This may reflect a basic human condition and be necessary to provide a balance between being something objectifiable that exist here and now, and at the same time being in an ongoing process which is still open and undetermined. Incorporating these different aspects and understanding the dynamics between them may therefore have important implications for the design and interpretation of feedback measures. Clinicians working without a feedback system might also find exploring these topics with patients advantageous.

Employing a cognitive interviewing approach to investigate response processes represents a valuable and underutilized source of validity evidence [23]. Its integration into validation practices should therefore be prioritized in future research to enhance the interpretative justification of scores on self-report measures. The current study also demonstrates that exploration of response processes can be used to inform the further development and design of self-report measures. Although this study focused on improving clinical feedback systems, we consider the approach to have relevance for other practices as well, such as other forms of measurement-based care and digital health records.

### Strengths and limitations

A strength of this study is that it provides knowledge about how repeated measurement works in practice and how different items function together from the responder’s point of view. The use of cognitive interviewing to study interpretations and reasoning related to specific items is an untraditional but helpful approach to obtain a type of knowledge that is difficult to access with other approaches. The research successfully identified several critical factors, including motivation, goals, and subjective feelings of change, that influenced how patients respond to feedback measures. However, the generalizability of the findings has limitations that should be taken into consideration and addressed in future research. First, the items included in this study were limited to the Norse feedback system. The inclusion of items from other measures in future research could strengthen the generalizability of the findings and may also reveal other problems not identified in the current study. Second, as this study is based on a specific patient population and context, the results may not be directly transferable to other settings. Most of the participants in the current study were in recovery, which might be characterized by other response processes and needs compared to patients with more active substance abuse. Furthermore, although we recruited participants from both inpatient and outpatient treatment settings, they were all recruited from the same clinic. Later studies should therefore include participants from other treatment settings and other clinics to check transferability of findings.

## Conclusions

The findings of this study extend the knowledge base and the qualitative foundation for longitudinal monitoring of SUDs and have implications for how feedback measures should be designed. The findings also increase the knowledge about the process of change and highlight patient experiences that are worth paying attention to when tracking progress via feedback system in treatment of SUDs.

The study highlights the essential role of capturing a comprehensive range of treatment-relevant factors through feedback systems in treatment of SUDs. Our analysis reveals that effective feedback mechanisms must account for preliminary outcomes, patient attitudes toward the process of change, and the dynamic interplay between their passive and active roles in treatment. Additionally, integrating both objective measures and subjective experiences of change is crucial for accurately reflecting individual progress.

The findings indicate that patients interpret feedback items through their unique contexts, including their motivations, goals, and personal experiences. This personalized approach underscores the need for feedback systems to adapt to incorporate these varied dimensions effectively. By doing so, these systems can better address the complexities of the treatment process and provide more meaningful insights into patient progress.

## Data Availability

No datasets were generated or analysed during the current study.
